# Designing 2D Wide Bandgap Semiconductor B_12_X_2_H_6_ (X=O, S) Based on Aromatic Icosahedral B_12_

**DOI:** 10.3390/nano15231803

**Published:** 2025-11-29

**Authors:** Pei Gong, Jun-Hui Yuan, Gen-Ping Wu, Zhi-Hong Liu, Hao Wang, Jiafu Wang

**Affiliations:** 1 School of Mathematics and Physics, Nanyang Institute of Technology, Nanyang 473004, China; 2 School of Physics and Mechanics, Wuhan University of Technology, Wuhan 430070, China; 3 Wuhan Second Ship Design and Research Institute, Wuhan 430205, China

**Keywords:** two-dimensional materials, borophene, carrier mobility, semiconductor, first-principles calculations

## Abstract

Constructing two-dimensional (2D) novel materials using superatoms as building blocks is currently a highly promising research field. In this study, by employing an oxidation strategy and based on first-principles calculations, we successfully predicted two types of 2D borides, namely B_12_X_2_H_6_ (X=O, S), with icosahedral B_12_ serving as their core structural unit. Ab initio molecular dynamics simulations demonstrated that these two borides exhibit exceptionally high structural stability, retaining their original structural characteristics even under extreme temperature conditions as high as 2200 K. Electronic structure calculations revealed that B_12_O_2_H_6_ and B_12_S_2_H_6_ are both wide-bandgap indirect semiconductors, with bandgap widths reaching 4.92 eV and 5.25 eV, respectively. Analysis via deformation potential theory showed that the phonon-limited carrier mobilities of B_12_X_2_H_6_ can reach up to 1469 cm^2^V^−1^s^−1^ (for B_12_O_2_H_6_) and 635 cm^2^V^−1^s^−1^ (for B_12_S_2_H_6_). Notably, the surfaces of B_12_X_2_H_6_ demonstrate excellent migration performance for alkali metal ions, with migration barriers as low as 0.15 eV (for B_12_O_2_H_6_) and 0.033 eV (for B_12_S_2_H_6_). This study not only expands the family of 2D materials based on B_12_ superatoms but also provides a solid theoretical foundation for the potential application of B_12_X_2_H_6_ in the field of low-dimensional materials.

## 1. Introduction

Boron, as a unique element located at the junction of metals and non-metals in the periodic table, possesses electronic structural characteristics akin to a magical key, opening a gateway for boron-based materials to a realm of physicochemical diversity far surpassing that of traditional two-dimensional (2D) materials. In 2015, the teams of Tai et al. [1] and Mannix et al. [2] successfully synthesized 2D boron monolayers on copper foil and silver substrates, respectively. This milestone achievement not only robustly confirmed the stable existence of boron atomic layers at room temperature but also heralded a new era in 2D borophene research, guiding researchers into this field full of unknowns and surprises [3].

Boron’s electron-deficient nature renders it akin to a masterful architect, inclined to construct multicenter bonding networks. This characteristic blossoms brilliantly at the 2D scale, giving rise to a rich array of structural polymorphisms [4,5,6]. Theoretical calculations, like a precise prophet, have unveiled that borophene may exist in over 20 stable configurations, including α-, β-, and γ-phase [7,8,9]. Among them, the γ-phase boron monolayer, through the synergistic interplay of B_12_ and B_2_ units, exhibits remarkable mechanical strength with a Young’s modulus of up to 398 GPa and exceptional thermal stability, maintaining structural integrity even at 1000 °C [10]. However, the non-layered structure of bulk boron poses a formidable barrier, rendering traditional mechanical exfoliation methods ineffective. To overcome this challenge, researchers have continuously developed novel preparation techniques, such as chemical vapor deposition (CVD) and molecular beam epitaxy (MBE) [11,12]. Nevertheless, constrained by substrate dependency, the path to large-scale preparation remains fraught with challenges.

Leveraging its flexibly adjustable electronic properties, boron can give rise to numerous superatoms. Among them, the experimentally known most stable superatom, the icosahedral borane B_12_H_12_^2−^, stands as one of its outstanding masterpieces [13]. A growing body of experimental and theoretical evidence supports that in the “bottom-up” approach, cluster superatoms, akin to exquisite building blocks, can serve as construction units to erect the grand edifice of self-assembled compounds and nanomaterials [14,15,16]. It is widely recognized that all 17 experimentally observed bulk boron allotropes to date contain icosahedral B_12_ structural units. In most cases, these units are accompanied by a certain number of boron atoms located outside the cage as interstitial atoms [17,18]. Therefore, constructing 2D borophene based on icosahedral B_12_ undoubtedly represents a field of immense research value and potential. Recently, Yan et al., utilizing aromatic icosahedral superatoms *I*_h_ B_12_H_12_^2−^ and *D*_5d_ 1,12-C_2_B_10_H_12_ as construction units, predicted a series of core–shell polyhedral boranes and carboranes through a clever “bottom-up” approach with the aid of density functional theory calculations [19]. These findings can be further extended to form 1D and 2D boranes, injecting new vitality into borophene and borane research and significantly enriching the research landscape in this field.

**Figure 1:**
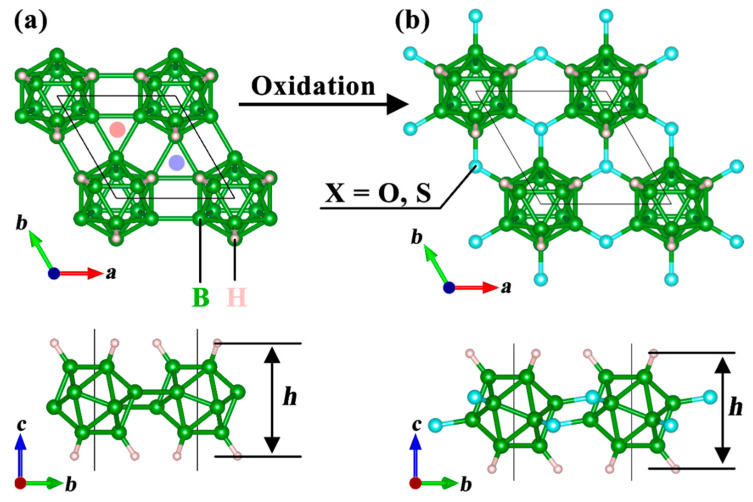
Crystal structure of monolayer (**a**) B_12_H_6_ and (**b**) B_12_X_2_H_6_ (X=O, S). The light red and light blue solid circles represent the oxidation sites corresponding to the bottom surface and the top surface of B_12_H_6_, respectively.

Among the materials proposed by Yan et al., the 2D B_12_H_6_ monolayer has attracted our particular attention. As illustrated in Figure 1a, B_12_H_6_ forms a stable 2D network through inter-unit B-B bonds. However, our analysis revealed a critical detail: the B-B bond length connecting adjacent B_12_H_6_ units is 2.004 Å, notably longer than the typical B-B bond lengths observed in conventional borophene. This longer bond indicates relatively weaker structural stability but also presents a valuable opportunity for material modification. For instance, introducing oxidation at the B-containing triangular sites at the center of the B_12_H_6_ unit (marked by light red and light blue solid circles in Figure 1a) could yield a new class of borane-derived materials (Figure 1b). This work is precisely based on the aforementioned design rationale, systematically investigating the stability, electronic structure, and surface ion migration characteristics of the B_12_H_6_ oxidation-derived system B_12_X_2_H_6_ (X=O, S). The detailed findings of this study are presented in Section 3.

## 2. Methods

The calculations in this study were carried out utilizing the projector augmented-wave (PAW) method [20,21] as implemented in the VASP [22,23]. The valence electron configuration of H, B, O, S, Li, Na and K were configured as 1*s*^1^, 2*s*^2^2*p*^1^, 2*s*^2^2*p*^4^, 3*s*^2^3*p*^4^, 2*s*^1^, 3*s*^1^ and 3*p*^6^4*s*^1^. The plane wave cutoff energy was fixed at 500 eV. For the exchange-correlation energy, the generalized gradient approximation (GGA) in the Perdew-Burke-Ernzerhof (PBE) formulation [24] was adopted. Considering the well-known issue of band gap underestimation inherent in GGA [25,26], the screened hybrid functional HSE06 was further employed for electronic band structure calculations [27]. During structural optimization, stringent criteria were applied, including a force threshold of 0.005 eV/Å in any direction, an energy criterion of 10^−7^ eV, and a dense *k*-mesh (13 × 13 × 1, Γ-centered). To prevent interactions between adjacent layers, a vacuum slab of up to 20 Å was introduced along the z-direction for 2D B_12_X_2_H_6_. To assess the thermodynamic stability of 2D B_12_X_2_H_6_, ab initio molecular dynamics (AIMD) simulations [28] were conducted using a 3 × 3 × 1 supercell (comprising a total of 180 atoms for each 2D B_12_X_2_H_6_ structure) over a duration of 5 ps at various temperatures. Phonon dispersion calculations, based on density functional perturbation theory, were performed using Phonopy [29]. The correction for van der Waals forces between adsorbed ions and the substrate during ion migration was implemented using DFT-D3 [30]. For data analysis, the VASPKIT [31] and VESTA [32] software packages were utilized.

## 3. Results and Discussion

### 3.1. Structure and Stability

Figure 1 presents the top and side views of the crystal structures of B_12_H_6_ (Figure 1a) and its derivative material, B_12_X_2_H_6_ (Figure 1b). The crystal symmetry of B_12_X_2_H_6_ remains consistent with that of B_12_H_6_; however, the introduction of the X element induces lattice expansion. Specifically, the lattice constant increases from 4.898 Å in B_12_H_6_ to 5.409 Å in B_12_O_2_H_6_ and 5.950 Å in B_12_S_2_H_6_, with detailed data provided in Table 1. During this structural evolution, the bond lengths of B-H exhibit minimal variation (1.181 Å in B_12_H_6_, 1.189 Å in B_12_O_2_H_6_, and 1.192 Å in B_12_S_2_H_6_), while the bond lengths of B-B within the boron icosahedron also remain relatively stable (1.745–1.793 Å in B_12_H_6_, 1.764–1.776 Å in B_12_O_2_H_6_, and 1.777–1.797 Å in B_12_S_2_H_6_). In contrast, the bond lengths of B-O and B-S in B_12_O_2_H_6_ and B_12_S_2_H_6_ stabilize at 1.497 Å and 1.894 Å, respectively. Another notable structural change is observed in the layer thickness (h), where the layer thickness of B_12_X_2_H_6_ is consistently smaller than that of B_12_H_6_ (4.674 Å), and the layer thicknesses of B_12_O_2_H_6_ and B_12_S_2_H_6_ are nearly identical (4.609 Å and 4.604 Å, respectively). Overall, except for the significant lattice expansion, the changes in other structural parameters before and after oxidation are relatively limited.

**Figure 2:**
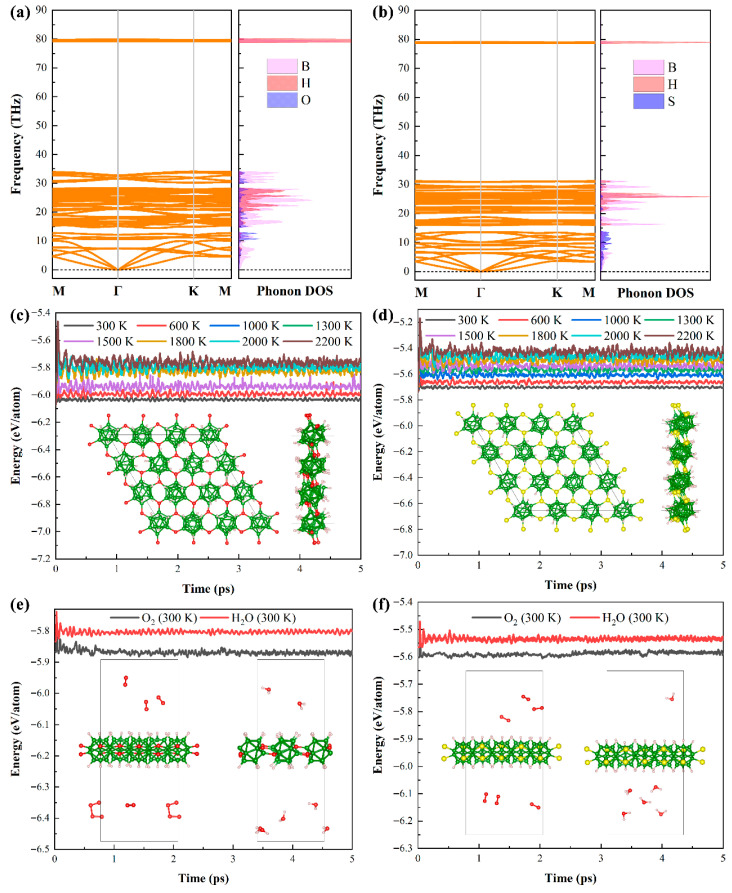
Phonon dispersion and density of states of (**a**) B_12_O_2_H_6_ and (**b**) B_12_S_2_H_6_. The AIMD results of (**c**) B_12_O_2_H_6_ and (**d**) B_12_S_2_H_6_ under various simulation temperatures, respectively. The AIMD results of (**e**) B_12_O_2_H_6_ and (**f**) B_12_S_2_H_6_ under O_2_ and H_2_O environment at 300 K. The insert is the final crystal structure of B_12_X_2_H_6_ at 2200 K and 300 K (for O_2_ and H_2_O) at the end of simulation time.

From a design strategy perspective, two novel derivatives (B_12_O_2_H_6_ and B_12_S_2_H_6_) can be successfully obtained via the oxidation of B_12_H_6_. Nevertheless, to verify their experimental feasibility, a systematic evaluation of their stability remains necessary. The primary focus is on kinetic stability, as shown in Figure 2a,b. Phonon spectrum analysis results, based on DFPT, show no imaginary frequencies for either material, which indicates excellent kinetic stability. Meanwhile, the frequencies of the phonon spectra are primarily distributed in two regions: one ranging from 0 to 35 THz, where the phonon density of states (DOS) is jointly contributed by B, X, and H elements; the other is near 80 THz, where the phonon DOS is mainly contributed by B and H elements. The phonon spectrum results further confirm the high strength of the B-H bond. Subsequently, we systematically simulated the thermal stability of B_12_X_2_H_6_ at different temperatures, as presented in Figure 2c,d. The simulation results show minimal total energy fluctuations in B_12_X_2_H_6_ under low-temperature conditions, which demonstrates its excellent resistance to thermal perturbations. As the simulation temperature increases, the fluctuations in total energy gradually intensify. Combined with the analysis of the crystal structure at the end of the simulation, it is found that even at a high temperature of 2200 K, B_12_X_2_H_6_ can still maintain a stable structure (without structural collapse), which fully proves its excellent high-temperature stability and lays a solid foundation for the application of B_12_X_2_H_6_ in high-temperature fields. Moreover, we further conducted an in-depth assessment of the stability of B_12_X_2_H_6_ in O_2_ and H_2_O environments under both room temperature (300 K) and high temperature (1000 K) conditions. The relevant assessment results are presented in Figure 2e,f and Figure S1, respectively. The findings show that at room temperature, neither O_2_ nor H_2_O chemically reacts with the surface of B_12_X_2_H_6_—a phenomenon that fully confirms the material’s excellent environmental stability. However, when the temperature increases to 1000 K, the scenario changes: B_12_X_2_H_6_ undergoes dehydrogenation when exposed to an H_2_O environment (Figure S1), yet it still maintains excellent structural stability in an O_2_ environment. Based on this, in practical application scenarios, no additional protective measures are required under low-temperature conditions; but under high-temperature conditions, effective control of environmental humidity is still necessary. Subsequently, the mechanical stability of B_12_X_2_H_6_ was evaluated based on independent elastic constants, and the specific results are listed in Table 2. The evaluation results show that B_12_X_2_H_6_ fully meets the Born-Huang criteria [33], indicating good mechanical stability. The aforementioned stability analysis results fully confirm that B_12_X_2_H_6_ exhibits excellent performance in terms of kinetic, thermal, environmental, and mechanical stability, which undoubtedly provides a solid theoretical foundation for its experimental synthesis and potential applications. Regarding experimental preparation, some superatomic systems can currently be synthesized via CVD and MBE techniques [34]. Based on this, and following scientific reasoning, we tentatively propose that the two borides designed in this work are also highly likely to be compatible with these two preparation methods

**Figure 3:**
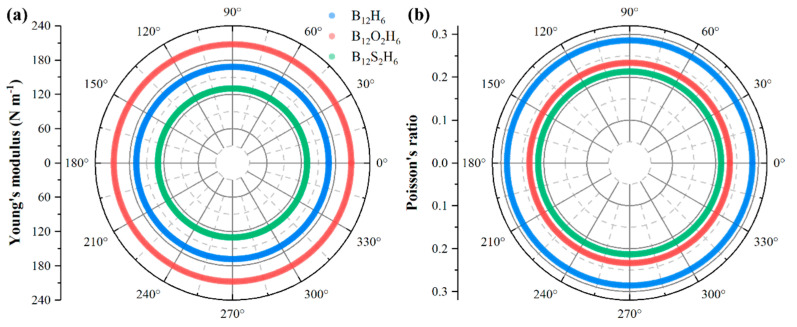
The calculated angle-dependent (**a**) Young’s modulus and (**b**) Poisson ratio of B_12_H_6_ and B_12_X_2_H_6_.

Furthermore, based on the obtained independent elastic constants, we calculated the angle-dependent Young’s modulus (*Y*) and Poisson’s ratio (*v*) of B_12_X_2_H_6_. As shown in Figure 3, both the Young’s modulus and Poisson’s ratio of B_12_H_6_ and B_12_X_2_H_6_ exhibit isotropic behavior. Among these materials, B_12_O_2_H_6_ has the highest Young’s modulus (207.45 N m^−1^), followed by B_12_H_6_ (168.13 N m^−1^), while B_12_S_2_H_6_ has the lowest Young’s modulus at 130.31 N m^−1^. As a direct indicator of material stiffness, Young’s modulus show that among the three materials, B_12_O_2_H_6_ has the highest stiffness, whereas B_12_S_2_H_6_ has the lowest. This also indirectly reflects differences in the strength of chemical bonds within the materials. In terms of Poisson’s ratio, both B_12_O_2_H_6_ (0.234) and B_12_S_2_H_6_ (0.213) are smaller than the their parent material B_12_H_6_ (0.286) and are comparable to that of MoS_2_ (0.21) [4]. Furthermore, the Young’s modulus of B_12_X_2_H_6_ is lower than that of graphene (~340 N m^−1^) [35] and is comparable to or slightly lower than that of MoS_2_ (~200 N m^−1^) [36].

### 3.2. Electronic Properties

**Figure 4:**
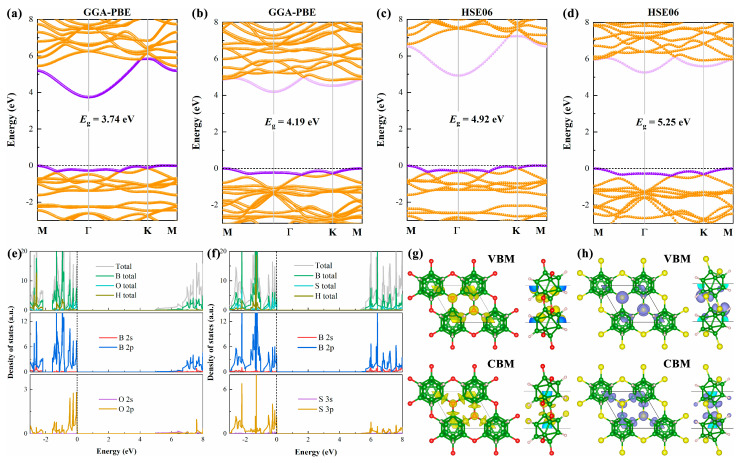
Calculated electronic band structures of (**a**,**c**) B_12_O_2_H_6_ and (**b**,**d**) B_12_S_2_H_6_ at GGA-PBE and HSE06 level. Projected density of states of (**e**) B_12_O_2_H_6_ and (**f**) B_12_S_2_H_6_ at HSE06 level. The corresponding VBM and CBM of (**g**) B_12_O_2_H_6_ and (**h**) B_12_S_2_H_6_. The isosurface is set to 0.04 *e* Å^−3^.

Subsequently, we focused our research on the electronic structure of B_12_X_2_H_6_, with relevant details illustrated in Figure 4. As clearly observed from Figure 4a,b, both B_12_O_2_H_6_ and B_12_S_2_H_6_ are wide-bandgap indirect semiconductors. Specifically, at the GGA-PBE level, the bandgap value of B_12_O_2_H_6_ is 3.74 eV, while that of B_12_S_2_H_6_ is even larger, reaching 4.19 eV. After hybrid functional correction (HSE06), the bandgaps of both compounds further increase: B_12_O_2_H_6_ and B_12_S_2_H_6_ exhibit bandgaps of 4.92 eV and 5.25 eV, respectively. These values are comparable to those of well-studied wide-bandgap semiconductors such as Ga_2_O_3_ (~4.9 eV) [37,38]. Compared to the parent material B_12_H_6_ (1.552 eV at the GGA-PBE level and 2.210 eV at the HSE06 level), the bandgaps of B_12_X_2_H_6_ are significantly larger. This phenomenon indicates that the introduction of O and S elements further enhances the localization of electrons within the system, thereby increasing the bandgap.

Further analysis shows that the conduction band minima (CBMs) of both B_12_O_2_H_6_ and B_12_S_2_H_6_ are located at the Γ point, while the positions of their valence band maxima (VBMs) differ. Specifically, the VBM of B_12_O_2_H_6_ is approximately centered between the K and M points while that of B_12_S_2_H_6_ is located at the M point. Furthermore, compared to the conduction band, the valence band of B_12_X_2_H_6_ near the Fermi level shows weaker dispersion, implying larger electron density of states (DOS) or carrier effective masses. Figure 4e,f display the partial density of states (PDOS) of B_12_O_2_H_6_ and B_12_S_2_H_6_, respectively, calculated using the HSE06 functional. Consistent with the band structure characteristics, the electron density of states of B_12_X_2_H_6_ in the valence band region is relatively high near the Fermi level, and to some extent, it approaches the DOS characteristics of the “Mexican hat”-type band structure. A high valence band DOS is beneficial for carrier accumulation, which in turn enhances the performance of electronic devices. Further analysis indicates that valence band DOS is mainly contributed by the B-2p and X-2p/3p (O-2p, S-3p) orbitals, highlighting the significant impact of X element (O/S) introduction on the system’s electronic structure. Finally, we also present the spatial charge distributions corresponding to the VBM and CBM of B_12_O_2_H_6_ and B_12_S_2_H_6_, as shown in Figure 4g,h. The results demonstrate that in B_12_X_2_H_6_, the charge corresponding to the VBM is primarily localized on the X atoms and the B atoms bonded to the X atoms. For the CBM, in addition to the charge localized on the X atoms and the B atoms bonded to the X atoms, there is also some charge localized on the H atoms.

**Figure 5:**
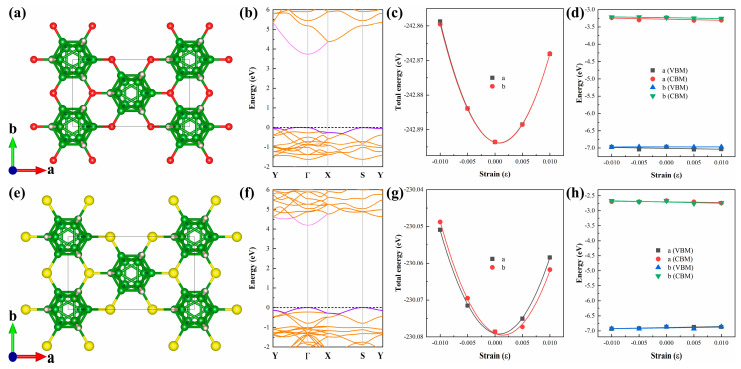
(**a**) The orthorhombic cell and (**b**) its energy band corresponding to B_12_O_2_H_6_. The fitting curves of (**c**) the in-plane elastic modulus *C*_2D_ and (**d**) the deformation potential constant *E*_1_ of B_12_O_2_H_6_ along *a* and *b* directions, respectively. (**e**) The orthorhombic cell and (**f**) its energy band corresponding to B_12_S_2_H_6_. The fitting curves of (**g**) the in-plane elastic modulus *C*_2D_ and (**h**) the deformation potential constant *E*_1_ of B_12_S_2_H_6_ along *a* and *b* directions, respectively.

For wide-bandgap semiconductors like B_12_X_2_H_6_, carrier mobility is another key parameter of interest. Based on deformation potential theory [39], we used the orthorhombic cells of B_12_X_2_H_6_ to calculate their electronic structures, in-plane elastic constants, and deformation potential constants. The relevant results are shown in Figure 5. By fitting the band structures, we obtained the effective masses of electrons and holes for B_12_X_2_H_6_ along the a and b directions. The specific data are presented in Table 3. For B_12_O_2_H_6_, the effective masses of electrons and holes along the a direction differ significantly. The electron effective mass is as high as 11.05 *m*_0_, while the hole effective mass is only 0.58 *m*_0_, indicating obvious anisotropy. This anisotropy is mainly attributed to the relatively weak valence band dispersion along the a direction. In contrast, B_12_O_2_H_6_ shows a smaller difference in effective masses along the b direction: the hole effective mass is 0.72 *m*_0_, and the electron effective mass is 2.00 *m*_0_. In B_12_S_2_H_6_, the effective masses of holes and electrons along the a and b directions are 1.99 *m*_0_ and 1.55 *m*_0_, respectively, while those along the b direction are 1.51 *m*_0_ and 0.46 *m*_0_, respectively. Thus, B_12_S_2_H_6_ exhibits less anisotropy than B_12_O_2_H_6_.

Finally, we combined the deformation potential constants and in-plane elastic constants to calculate the carrier mobilities of B_12_O_2_H_6_ and B_12_S_2_H_6_, with specific data presented in Table 3. The hole and electron mobilities of B_12_O_2_H_6_ along the a direction are 20.71 and 672.99 cm^2^V^−1^s^−1^, respectively, while those along the b direction are extremely high (197,818.31 cm^2^V^−1^s^−1^ for holes and 340.61 cm^2^V^−1^s^−1^ for electrons). The extremely high hole mobility of B_12_O_2_H_6_ along the b direction is mainly attributed to its extremely small fitted deformation potential constant (0.10 eV). However, such a large anisotropy is clearly inconsistent with physical reality. Thus, we re-estimated the hole mobility of B_12_O_2_H_6_ using the anisotropy correction method for carrier mobility in 2D materials proposed by Lang et al. [40] (see the underlined and bolded results in Table 3). After correction, the hole mobility of B_12_O_2_H_6_ along the b direction is 1469.41 cm^2^V^−1^s^−1^. For B_12_S_2_H_6_, the hole mobility is approximately 50 cm^2^V^−1^s^−1^, while the electron mobility is higher (up to 450 cm^2^V^−1^s^−1^). According to Lang et al.’s [40] method, the electron mobility ranges from 250 to 630 cm^2^V^−1^s^−1^. In summary, the carrier mobility of B_12_X_2_H_6_ is not outstanding among 2D materials, being much lower than that of 2D materials with ultra-high carrier mobilities (e.g., black phosphorus [41]). However, their carrier mobility is comparable to or even higher than that of MoS_2_ (~200 cm^2^V^−1^s^−1^) [42]. Additionally, it should be noted that in this work, we did not consider the influence of defects or impurities on the electronic structure and carrier mobility of the B_12_X_2_H_6_ monolayer. However, these factors play a non-negligible role in practical applications. Thus, when applying these findings to practical scenarios, these factors need to be comprehensively considered.

### 3.3. Alkali Ions Migration

**Figure 6:**
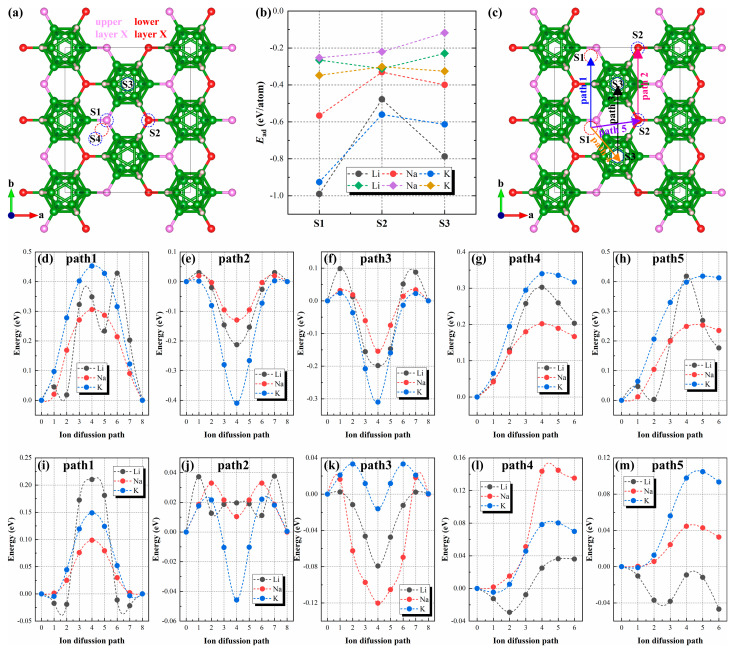
(**a**) Schematic diagram of ion adsorption sites on B_12_X_2_H_6_. The X atoms in the upper layer (light purple) and the lower layer (red) are marked with different colors, respectively. (**b**) The adsorption energy of alkali metal ions on B_12_X_2_H_6_. The solid circles and diamonds represent the corresponding results for B_12_O_2_H_6_ and B_12_S_2_H_6_, respectively. (**c**) Schematic representation of the ion migration paths of ions on B_12_X_2_H_6_. Calculated migration energy barrier of Li/Na/K ions on B_12_O_2_H_6_ along (**d**) path 1, (**e**) path 2, (**f**) path 3, (**g**) path 4, and (**h**) path 5. Calculated migration energy barrier of Li/Na/K ions on B_12_S_2_H_6_ along (**i**) path 1, (**j**) path 2, (**k**) path 3, (**l**) path 4, and (**m**) path 5.

Last but not least, we investigated the migration behavior of alkali metal ions (Li, Na and K) on the surface of B_12_X_2_H_6_. Figure 6a illustrates the supercell of the orthorhombic structure of B_12_X_2_H_6_. Based on symmetry analysis, four inequivalent adsorption sites exist on its surface, namely above the upper-layer X atoms (denoted as S1), above the lower-layer X atoms (S2), above the B_12_ cluster (S3), and at the hollow sites between adjacent B_12_X_2_H_6_ units (S4). After optimizing the adsorption structures of alkali metal ions at these four sites, we found that the alkali metal ions at the S1 and S4 sites eventually overlapped, with the final position closer to the S1 site (marked by the red dashed box in Figure 6a). Therefore, only the three sites, S1, S2, and S3, were considered in subsequent studies. Subsequently, we calculated the adsorption energies of alkali metal ions at these three sites (Figure 6b). The results showed that, except for Na on the B_12_S_2_H_6_ surface (where the lowest adsorption energy site was S2), the lowest adsorption energy site for all other adsorption system is S1. Furthermore, the adsorption energies of alkali metal ions on the B_12_O_2_H_6_ surface were significantly higher than those on the B_12_S_2_H_6_ surface: the former ranges from −0.99 to −0.57 eV/atom, and the latter ranges from −0.35 to −0.25 eV/atom. Lower adsorption energies correspond to weaker interactions, which significantly influence the surface migration behavior of ions. Based on the adsorption sites calculation results, we designed five migration pathways: path1 (S1→S1), path2 (S2→S2), path3 (S3→S3), path4 (S1→S3), and path5 (S1→S2). The migration energy barriers were calculated using the CI-NEB method [43], with the results presented in Figure 6d–m.

The migration energy barrier calculations for alkali metal ions on the B_12_O_2_H_6_ surface reveal that the strong adsorption interaction between alkali metal ions and the B_12_O_2_H_6_ surface results in relatively high migration energy barriers. Specifically, the migration energy barriers for K ions on the B_12_O_2_H_6_ are relatively high (0.31 to 0.45 eV), while those for Na ions are the lowest among the three ions (0.15 to 0.31 eV); and those for Li ions ranges from 0.24 to 0.43 eV. In contrast, the migration energy barriers for alkali metal ions on B_12_S_2_H_6_ are significantly lower than those on B_12_O_2_H_6_, which is attributable to the lower adsorption energies of alkali metal ions on B_12_S_2_H_6_. The specific values are as follows: 0.04 to 0.23 eV for Li ions, 0.033 to 0.15 eV for Na ions, and 0.05 to 0.15 eV for K ions. These results indicated that alkali metal ions exhibited relatively fast migration rates on B_12_O_2_H_6_, with migration energy barriers comparable to those of materials such as graphene (0.277 eV) [44], MoS_2_ (0.21 eV) [45], and β-B_12_ (0.3 eV) [46]. On B_12_S_2_H_6_, the migration rates are even faster, with minimum migration energy barriers comparable to those of materials such as Si_3_C (0.18 eV) [47], C_3_N (0.07 eV) [48], and PdI_2_ (0.08 eV) [49]. The excellent alkali ion migration performance of B_12_X_2_H_6_ suggests its potential applications in fields such as energy storage and chemical sensing.

## 4. Conclusions

In summary, using first-principles calculations and an oxidation strategy, we have successfully designed two novel two-dimensional (2D) borides, B_12_X_2_H_6_ (X=O, S). The core structural unit of these two borides is icosahedral boranes with partial hydrogen deficiency, and both materials exhibit exceptional kinetic, thermal, mechanical, and environmental stability. Notably, they can maintain excellent structural integrity even when exposed to an extreme high temperature of 2200 K. Hybrid functional calculations reveal that both B_12_O_2_H_6_ and B_12_S_2_H_6_ are wide-bandgap indirect semiconductors, with bandgap of 4.92 eV and 5.25 eV, respectively. Further calculations using the modified deformation potential theory indicate that the mobility of B_12_O_2_H_6_ ranges from 44 to 1469 cm^2^V^−1^s^−1^, while that of B_12_S_2_H_6_ ranges from 47 to 635 cm^2^V^−1^s^−1^. In-depth analysis shows that the relatively low carrier mobility of B_12_X_2_H_6_ is mainly attributed to the large effective masses and deformation potential constant of the carriers. Moreover, due to the low adsorption energy of alkali metal ions on the B_12_X_2_H_6_ surface, B_12_X_2_H_6_ exhibits excellent alkali metal ion transport performance, with migration energy barriers ranging from 0.15 to 0.45 eV (for B_12_O_2_H_6_) and from 0.033 to 0.23 eV (for B_12_S_2_H_6_). The findings of this study lay a solid theoretical foundation for the potential application of B_12_X_2_H_6_, and provide guidance for subsequent research on B_12_-based 2D materials.

## Figures and Tables

**Table 1:** Calculated lattice constants *a*/*b* (Å), bond length *l* (Å), layer thickness *h* (Å), and band gaps (at GGA-PBE and HSE06 level, eV) of B_12_H_6_ and B_12_X_2_H_6_.

Materials	*a*/*b*	*l*_B-H_	*l*_B-B_	*l*_B-B/X_	*h*	$E_{g}^{PBE}$	$E_{g}^{HSE}$
B_12_H_6_	4.898	1.181	1.745~1.793	2.004	4.674	1.552	2.210
B_12_O_2_H_6_	5.409	1.189	1.764~1.776	1.497	4.609	3.746	4.927
B_12_S_2_H_6_	5.950	1.192	1.777~1.797	1.894	4.604	4.196	5.257

**Table 2:** Calculated elastic constant *C*_11_, *C*_22_, *C*_12_, *C*_66_ (N m^−1^), axis Young’s modulus (*Y*_11_/*Y*_22_) and Poisson’s ratio (*v*_11_/*v*_22_) of B_12_O_2_H_6_ and B_12_S_2_H_6_.

Materials	*C*_11_/*C*_22_	*C*_12_	*C*_66_	*Y*_11_/*Y*_22_	*v*_11/_ *v*_22_
B_12_H_6_	183.09	52.34	65.38	168.13	0.286
B_12_O_2_H_6_	219.41	51.23	84.09	207.45	0.234
B_12_S_2_H_6_	136.51	29.08	53.71	130.31	0.213

**Table 3:** The calculated effective mass (m*, *m*_0_) of electron (*e*) and hole (*h*), in-plane elastic modulus *C*_2D_ (N m^−1^), deformation potential constant (*E*_1_, eV) and corresponding carrier mobility (*μ*, cm^2^V^−1^s^−1^) of B_12_O_2_H_6_ and B_12_S_2_H_6_ monolayers. The carrier mobility results based on the BS method and Lang et al.’s [40] correction method (bold and underlined) are provided separately.

Materials	Carrier Type	x-Axis				y-Axis			
		*m*_a_^*^	${|E}_{1a}|$	*C*_2D_	*μ*_x_	*m*_b_^*^	${|E}_{1b}|$	*C*_2D_	*μ*_y_
B_12_O_2_H_6_	h	11.05	2.51	190.92	20.71 /**44.40**	0.72	0.10	188.63	197,818.31 /**1469.41**
	e	0.58	3.11	190.92	672.99/ **810.54**	2.00	2.34	188.63	340.61 /**269.75**
B_12_S_2_H_6_	h	1.99	3.45	127.16	49.24/ **57.49**	1.55	2.71	123.97	57.12 /**47.29**
	e	1.51	2.18	127.16	452.82 /**258.41**	0.46	3.94	123.97	443.64 /**635.46**

## Data Availability

All data needed to evaluate the conclusions in the paper are present in the paper.

## Supplementary Materials

The following supporting information can be downloaded at: https://www.mdpi.com/article/10.3390/nano15231803/s1, Figure S1: The AIMD results of B_12_X_2_H_6_ under O_2_ and H_2_O environment at 1000 K.
