# Supplementary material for: Designing 2D Wide Bandgap Semiconductor B12X2H6 (X=O, S) Based on Aromatic Icosahedral B12
Source: Nanomaterials (Basel). 2025 Nov 29;15(23):1803. doi: 10.3390/nano15231803 (PMC12693634; doi:10.3390/nano15231803)
Supplement: Supplementary file 1 [file nanomaterials-15-01803-s001.zip › nanomaterials-3995136-supplementary.pdf]

Supporting Information for

Designing 2D wide bandgap semiconductor $B_{12}X_2H_6$ (X=O, S) based on aromatic icosahedral B_{12}

Pei Gong[1], Jun-Hui Yuan[2,*], Gen-Ping Wu[3], Zhi-Hong Liu[3], Hao Wang[3,*] , and Jiafu Wang[2]

[1]School of Mathematics and Physics, Nanyang Institute of Technology, Nanyang 473004, China

[2]School of Physics and Mechanics, Wuhan University of Technology, Wuhan 430070, China

[3]Wuhan Second Ship Design and Research Institute, Wuhan 430205, China

[*]**Corresponding Author**

E-mail: yuanjh90@163.com (J.-H. Yuan); whuwanghao@163.com (H. Wang)

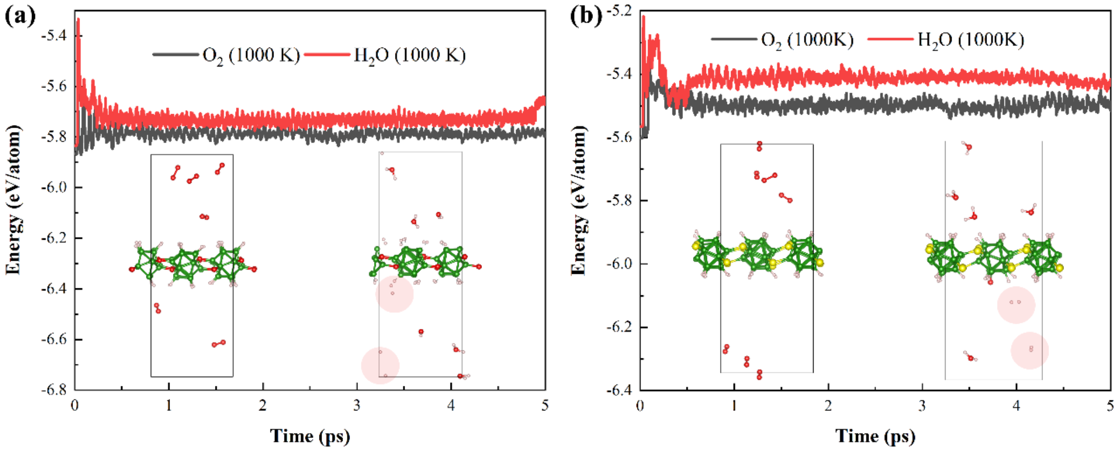

Figure S1. The AIMD results of (d) $B_{12}O_2H_6$ and (c) $B_{12}S_2H_6$ under O_2 and H_2O environment at 1000 K. The insert is the final crystal structure of $B_{12}X_2H_6$ under O_2 and H_2O environment at 1000 K at the end of simulation time.
